# Van der Pol model in two-delay differential equation representation

**DOI:** 10.1038/s41598-022-06911-3

**Published:** 2022-02-21

**Authors:** M. A. Elfouly, M. A. Sohaly

**Affiliations:** Department of Mathematics, Faculty of Science, Mansoura University, Mansoura, Egypt

**Keywords:** Diseases, Neurology, Engineering, Physics

## Abstract

The Van der Pol equation is the mathematical model of a second-order ordinary differential equation with cubic nonlinearity. Several studies have been adding time delay to the Van der Pol model. In this paper, the differential equation of the Van der Pol model and the RLC (resistor–inductor–capacitor) circuit are deduced as a delay differential equation. The Van der Pol delay model contains two delays, which allows the re-use of its applications in the suggested equation. The Taylor series was used to deduce ordinary differential equations from the delay differential equations in the case of small delays. Also, the model for Parkinson's disease modification is described as the Van der Pol model. A numerical simulation of the delay differential equations has been done to show the different cases that the delay differential equations can express using the MATLAB program.

## Introduction

The Van der Pol oscillator is a classic example of self-oscillatory system and is now considered as very useful mathematical model that can be used in much more complicated and modified systems. RLC circuit is an electrical circuit consisting of a resistor (R), an inductor (L), and a capacitor (C), ordinary differential equation (ODE) that expresses the LRC circuit if they are connected in series as damped harmonic oscillator:

1 $$ \frac{{{\text{d}}^{{2}} {\text{x}}}}{{{\text{dt}}^{{2}} }} + {\upmu }\frac{{{\text{dx}}}}{{{\text{dt}}}} + {\upbeta {\text{x}}} = {0} $$

In the 1920's by Balthazar van der Pol, this circuit is an RLC loop, but with the passive resistor of Ohm's Law replaced by triode^1^, the Dutch engineer Balthazar van der Pol presented a mathematical model to describe oscillations in electrical circuits. Balthazar van der Pol used ordinary differential equation as follows:

2 $$ \frac{{{\text{d}}^{{2}} {\text{x}}}}{{{\text{dt}}^{{2}} }} + {\upmu }\left( {{\text{x}}^{{2}} - {1}} \right)\frac{{{\text{dx}}}}{{{\text{dt}}}} + {\upbeta {\text{x}}} = {0} $$

Van der Pol is now regarded as the fundamental oscillation model in physical, electronic, biological, neurological, sociological, and economic terms^2^. Parkinson's disease was studied mathematically by G. Austin in 1961 and expressed the amplitude of hand tremor by a second-order differential equation, using the Van der Pol model^3^ in (2). Claudia (2009) expresses repetitive hand movements in Parkinson’s disease using a nonlinear two-delay differential equation^4^ as follows:

3 $$ \frac{{{\text{dx}}}}{{{\text{dt}}}} = {\text{a}}_{{1}} {\text{ x(t}} - {\uptau }_{{1}} {\text{) + a}}_{{2}} {\text{ x(t}} - {\uptau }_{{2}} {\text{) + a}}_{{3}} {\text{ x(t}} - {\uptau }_{{1}} {\text{ )x(t}} - {\uptau }_{{2}} {)} $$

The associated linear model for (3) is

4 $$ \frac{{{\text{dx}}}}{{{\text{dt}}}} = {\text{a}}_{{1}} {\text{ x(t}} - {\uptau }_{{1}} {)} + {\text{a}}_{{2}} {\text{ x(t}} - {\uptau }_{{2}} {)} $$

The effect of adding time delays to the Van der Pol model has been studied, such as Zakaria Ghouli's in^5^ study the presence of time delay in the electrical circuit of an excited van der Pol oscillator. In this paper, we derive the Van der Pol equation as a two-delay differential equation. We deduce the Van der Pol equation from the equation derived using Taylor series in the case of small delays. We use the deduced equation as a mathematical model for Parkinson's disease. We give some numerical studies that show the deduced equation expresses some cases of the Van der Pol equation, such as relaxation and oscillation.

## Represented RLC circuit as two-delay differential equation

In alternating current (AC) circuits with a capacitor the equation of the current intensity ${\text{I}}$ curve^6^ is given as:

5 $$ {\text{I}} = {\text{I}}_{{{\text{max}}}} \cos(2\uppi {\text{f t}}) $$

where, ${\text{f}}$ is the frequency, by differentiating with respect to time:

6 $$ \frac{{{\text{dI}}}}{{{\text{dt}}}} = - 2\uppi {\text{f I}}_{\max}\sin(2\uppi {\text{f t}}) $$

7 $$ \frac{{{\text{dI}}}}{{{\text{dt}}}} = - 2\uppi {\text{ f I}}_{\max} {\cos}\left( 2\uppi {\text{f}} \left( {\text{t}} - \frac{1}{{\text{4f}}} \right) \right) $$

8 $$ \frac{{{\text{dI}}}}{{{\text{dt}}}} = - 2\uppi {\text{f I}}({\text{t}} - \uptau_{1} )) $$

In AC circuits with an inductor the equation of the current intensity ${\text{I}}$ curve^6^ is given as:

9 $$ {\text{I}} = {\text{I}}_{\max} {\sin}(2\uppi {\text{f t}}) $$

where, ${\text{f}}$ is the frequency, by differentiating with respect to time we obtain,

10 $$ \frac{{{\text{dI}}}}{{{\text{dt}}}} = 2\uppi {\text{f I}}_{\max}  \cos(2\uppi {\text{f t}}) $$

11 $$ \frac{{{\text{dI}}}}{{{\text{dt}}}} = 2\uppi {\text{f I}}_{\max} \sin\left( 2\uppi {\text{f}}\left( {\text{t}} - \frac{3}{{\text{4f}}} \right) \right) $$

12 $$ \frac{{{\text{dI}}}}{{{\text{dt}}}} = 2\uppi {\text{f I}}({\text{t}} - \uptau _{2} )) $$

where, ${\uptau }_{{1}} = \frac{{1}}{{{\text{4f}}}}$ and ${\uptau }_{{2}} = \frac{{3}}{{{\text{4f}}}}$ are the time delays, then the model (4) is the delay differential equation for the RLC circuit. Approximated delay differential equations in (4) by using Taylor series expansion about zero delays, the Eq. (4) can be written as follows:

13 $$ \frac{{{\text{dx}}}}{{{\text{dt}}}} = {\text{a}}_{{1}} \, \left( {{\text{x}} - {\uptau }_{{1}} \frac{{{\text{dx}}}}{{{\text{dt}}}} + \frac{{{\uptau }_{{1}}^{{2}} }}{{2}}\frac{{{\text{d}}^{{2}} {\text{x}}}}{{{\text{d}}^{{2}} {\text{t}}}} + \cdots } \right) + {\text{a}}_{{2}} \, \left( {{\text{x}} - {\uptau }_{{2}} \frac{{{\text{dx}}}}{{{\text{dt}}}} + \frac{{{\uptau }_{{2}}^{{2}} }}{{2}}\frac{{{\text{d}}^{{2}} {\text{x}}}}{{{\text{d}}^{{2}} {\text{t}}}} + \cdots } \right) $$

14 $$ \left( {\frac{{{\text{a}}_{{1}} {\uptau }_{{1}}^{{2}} }}{{2}} + \frac{{{\text{a}}_{{2}} {\uptau }_{{2}}^{{2}} }}{{2}}} \right)\frac{{{\text{d}}^{{2}} {\text{x}}}}{{{\text{d}}^{{2}} {\text{t}}}} + \left( { - {\text{a}}_{{1}} {\uptau }_{{1}} - {\text{a}}_{{2}} {\uptau }_{{2}} - {1}} \right)\frac{{{\text{dx}}}}{{{\text{dt}}}} + \left( {{\text{a}}_{{1}} + {\text{a}}_{{2}} } \right){\text{x}} = {0} $$

15 $$ \frac{{{\text{d}}^{{2}} {\text{x}}}}{{{\text{d}}^{{2}} {\text{t}}}} - \frac{{{2(1} + {\text{a}}_{{1}} {\uptau }_{{1}} + {\text{a}}_{{2}} {\uptau }_{{2}} {)}}}{{{\text{a}}_{{1}} {\uptau }_{{1}}^{{2}} + {\text{a}}_{{2}} {\uptau }_{{2}}^{{2}} }}\frac{{{\text{dx}}}}{{{\text{dt}}}} + \frac{{{2}\left( {{\text{a}}_{{1}} + {\text{a}}_{{2}} } \right)}}{{{\text{a}}_{{1}} {\uptau }_{{1}}^{{2}} + {\text{a}}_{{2}} {\uptau }_{{2}}^{{2}} }}{\text{x}} = {0} $$

If $${\upmu } = - \frac{{{2(1} + {\text{a}}_{{1}} {\uptau }_{{1}} + {\text{a}}_{{2}} {\uptau }_{{2}} {)}}}{{{\text{a}}_{{1}} {\uptau }_{{1}}^{{2}} + {\text{a}}_{{2}} {\uptau }_{{2}}^{{2}} }}$$ and $${\upbeta } = \frac{{{2}\left( {{\text{a}}_{{1}} + {\text{a}}_{{2}} } \right)}}{{{\text{a}}_{{1}} {\uptau }_{{1}}^{{2}} + {\text{a}}_{{2}} {\uptau }_{{2}}^{{2}} }}$$.

Then we get the model (1)

### Stability analysis for the model in (4)

By substituting in (4) as ${\text{x(t) = e}}^{{{\uplambda {\text{t}}}}}$, then the characteristic equation is given as follow:

16 $$ \uplambda = {\text{a}}_{1} {\text{e}}^{ - \uplambda \tau_{1}}  + {\text{a}}_{2} {\text{ e}}^{ - \uplambda \tau _{2}}  $$

Delays and complex eigenvalues have effects on stability. If $\uplambda = \mu + {\text{i}}\omega$.

Therefore:

$$ \mu = {\text{a}}_{1} {\text{ e}}^{{ - \mu \tau_{1} }} \cos (\omega \tau_{1} ) + {\text{a}}_{2} {\text{ e}}^{{ - \mu \tau_{2} }} \cos (\omega \tau_{2} ), $$

$$ \omega = - {\text{a}}_{{1}} {\text{ e}}^{{ - \mu \tau_{{1}} }} \sin (\omega \tau_{{1}} {)} - {\text{a}}_{{2}} {\text{ e}}^{{ - \mu \tau_{{2}} }} \sin (\omega \tau_{{2}} {)}{\text{.}} $$

For very small delays ${\uptau }_{{1}} < < {1}\;{,}\;{\uptau }_{{2}} < < {1}$, the imaginary part $\omega \to 0$. Then the fixed point is asymptotically stable if,

17 $$ {\text{a}}_{{1}} + {\text{a}}_{{2}} \, < {0} $$

The fixed point for (15) is asymptotically stable if, $\frac{{{2(1} + {\text{a}}_{{1}} {\uptau }_{{1}} + {\text{a}}_{{2}} {\uptau }_{{2}} {)}}}{{{\text{a}}_{{1}} {\uptau }_{{1}}^{{2}} + {\text{a}}_{{2}} {\uptau }_{{2}}^{{2}} }} < 0$ and $\frac{{{2}\left( {{\text{a}}_{{1}} + {\text{a}}_{{2}} } \right)}}{{{\text{a}}_{{1}} {\uptau }_{{1}}^{{2}} + {\text{a}}_{{2}} {\uptau }_{{2}}^{{2}} }} > 0$^7^, so ${\text{a}}_{{1}} {\uptau }_{{1}}^{{2}} + {\text{a}}_{{2}} {\uptau }_{{2}}^{{2}} < 0$, ${1} + {\text{a}}_{{1}} {\uptau }_{{1}} + {\text{a}}_{{2}} {\uptau }_{{2}} > 0$ and ${\text{a}}_{{1}} + {\text{a}}_{{2}} \, < {0}$ which is the condition in (17) for very small delays.

## Represented Van der Pol equation

In the case of replacing resistance in an LRC circuit by a triode, the rate of change of current depends on the difference between ${\text{x(t}} - {\uptau }_{{1}} { )} - {\text{ x(t}} - {\uptau }_{{2}} { )}$.

The current of the anode is the difference between the cathode current and a grid current. From the special curves of the tripod, there is a phase difference between them and this amount can be multiplied by the square of the current because the relationship is non-linear^2^.

Thus, we get the following form:

18 $$ \frac{{\text{dx(t)}}}{{{\text{dt}}}} = {\text{ a}}_{{1}} {\text{ x(t}} - {\uptau }_{{1}} {\text{ ) + a}}_{{2}} {\text{ x(t}} - {\uptau }_{{2}} { )} - \left( {\text{x(t)}} \right)^{{2}} \left( {{\text{x(t}} - {\uptau }_{{2}} { )} - {\text{ x(t}} - {\uptau }_{{1}} { )}} \right) $$

Using Taylor series for small delays and mean value theory, the Eq. (9) can be written as follows:

19 $$ \frac{{{\text{dx}}}}{{{\text{dt}}}} = {\text{ a}}_{{1}} \, \left( {{\text{x}} - {\uptau }_{{1}} \frac{{{\text{dx}}}}{{{\text{dt}}}} + \frac{{{\uptau }_{{1}}^{{2}} }}{{2}}\frac{{{\text{d}}^{{2}} {\text{x}}}}{{{\text{d}}^{{2}} {\text{t}}}} + \cdots } \right) + {\text{a}}_{{2}} \, \left( {{\text{x}} - {\uptau }_{{2}} \frac{{{\text{dx}}}}{{{\text{dt}}}} + \frac{{{\uptau }_{{2}}^{{2}} }}{{2}}\frac{{{\text{d}}^{{2}} {\text{x}}}}{{{\text{d}}^{{2}} {\text{t}}}} + \cdots } \right) + \left( {{\uptau }_{{2}} - {\uptau }_{{1}} } \right){\text{x}}^{{2}} \frac{{{\text{dx}}}}{{{\text{dt}}}} $$

20 $$ \left( {\frac{{{\text{a}}_{{1}} {\uptau }_{{1}}^{{2}} }}{{2}} + \frac{{{\text{a}}_{{2}} {\uptau }_{{2}}^{{2}} }}{{2}}} \right)\frac{{{\text{d}}^{{2}} {\text{x}}}}{{{\text{d}}^{{2}} {\text{t}}}} + \left( { - {\text{a}}_{{1}} {\uptau }_{{1}} - {\text{a}}_{{2}} {\uptau }_{{2}} + {{(\uptau }}_{{2}} - {\uptau }_{{1}} {\text{)x}}^{{2}} - {1}} \right)\frac{{{\text{dx}}}}{{{\text{dt}}}} + \left( {{\text{a}}_{{1}} + {\text{a}}_{{2}} } \right){\text{x}} = {0} $$

21 $$ \frac{{{\text{d}}^{{2}} {\text{x}}}}{{{\text{d}}^{{2}} {\text{t}}}} + \frac{{{{2(\uptau }}_{{2}} - {\uptau }_{{1}} {)}}}{{{\text{a}}_{{1}} {\uptau }_{{1}}^{{2}} + {\text{a}}_{{2}} {\uptau }_{{2}}^{{2}} }}\left( {{\text{x}}^{{2}} - \frac{{{1} + {\text{a}}_{{1}} {\uptau }_{{1}} + {\text{a}}_{{2}} {\uptau }_{{2}} }}{{{\uptau }_{{2}} - {\uptau }_{{1}} }}} \right)\frac{{{\text{dx}}}}{{{\text{dt}}}} + \frac{{{2}\left( {{\text{a}}_{{1}} + {\text{a}}_{{2}} } \right)}}{{{\text{a}}_{{1}} {\uptau }_{{1}}^{{2}} + {\text{a}}_{{2}} {\uptau }_{{2}}^{{2}} }}{\text{x}} = {0} $$

If $${\upalpha } = \frac{{{1} + {\text{a}}_{{1}} {\uptau }_{{1}} + {\text{a}}_{{2}} {\uptau }_{{2}} }}{{{\uptau }_{{2}} - {\uptau }_{{1}} }}$$, $${\upmu } = \frac{{{{2(\uptau }}_{{2}} - {\uptau }_{{1}} {)}}}{{{\text{a}}_{{1}} {\uptau }_{{1}}^{{2}} + {\text{a}}_{{2}} {\uptau }_{{2}}^{{2}} }}$$ and $${\upbeta } = \frac{{{2}\left( {{\text{a}}_{{1}} + {\text{a}}_{{2}} } \right)}}{{{\text{a}}_{{1}} {\uptau }_{{1}}^{{2}} + {\text{a}}_{{2}} {\uptau }_{{2}}^{{2}} }}$$.

Then,

22 $$ \frac{{{\text{d}}^{{2}} {\text{x(t)}}}}{{{\text{d}}^{{2}} {\text{t}}}} + {\upmu }\left( {{\text{x}}^{{2}} - {\upalpha }} \right)\frac{{\text{dx(t)}}}{{{\text{dt}}}} + {\upbeta {\text{x}}} = {0} $$

Equation (12) is the Van der Pol equation. In general, the Van der Pol equation can be written as a delay differential equation with two small delays as follows:

23 $$ \frac{{\text{dx(t)}}}{{{\text{dt}}}} = {\text{ a}}_{{1}} {\text{ x(t}} - {\uptau }_{{1}} { )} + {\text{a}}_{{2}} {\text{ x(t}} - {\uptau }_{{2}} { )} - \left( {\text{x(t)}} \right)^{{2}} \left( {{\text{x(t}} - {\uptau }_{{2}} { )} - {\text{ x(t}} - {\uptau }_{{1}} { )}} \right) $$

where ${\text{a}}_{{1}}$ and ${\text{a}}_{{2}}$ are scalar parameters.

The critical points are for model in (23) is only zero fixed point. By linearization the model in (23) to analysis the stability:

$$ {\text{f(x,x(t}} - \tau_{{1}} { ),}\,{\text{x(t}} - \tau_{{2}} { ))} = {\text{ a}}_{{1}} {\text{ x(t}} - {\uptau }_{{1}} {\text{ ) + a}}_{{2}} {\text{ x(t}} - {\uptau }_{{2}} { )} - \left( {\text{x(t)}} \right)^{{2}} \left( {{\text{x(t}} - {\uptau }_{{2}} { )} - {\text{ x(t}} - {\uptau }_{{1}} { )}} \right) $$

$$ \left. {\frac{{\partial {\text{f(x(t),}}\,{\text{x(t}} - \tau_{{1}} { ),}\,{\text{x(t}} - \tau_{{2}} { ))}}}{{\partial {\text{x(t)}}}}} \right|_{{{\text{x(t)}} = {\text{x(t}} - \tau_{{1}} { )} = {\text{x(t}} - \tau_{{2}} { )} = {0}}} = \, 0 $$

$$ \left. {\frac{{\partial {\text{f(x(t),}}\,{\text{x(t}} - \tau_{{1}} { ),}\,{\text{x(t}} - \tau_{{2}} { ))}}}{{\partial {\text{x(t}} - \tau_{{1}} { )}}}} \right|_{{{\text{x(t)}} = {\text{x(t}} - \tau_{{1}} { )} = {\text{x(t}} - \tau_{{2}} { )} = {0}}} = {\text{a}}_{{1}} $$

$$ \left. {\frac{{\partial {\text{f(x(t),}}\,{\text{x(t}} - \tau_{{1}} { ),}\,{\text{x(t}} - \tau_{{2}} { ))}}}{{\partial {\text{x(t}} - \tau_{{2}} { )}}}} \right|_{{{\text{x(t)}} = {\text{x(t}} - \tau_{{1}} { )} = {\text{x(t}} - \tau_{{2}} { )} = {0}}} = {\text{a}}_{{2}} $$

The associated linear model for (23) is the model in (4). As explained previously the fixed point for (23) is asymptotically stable for very small delays ${\uptau }_{{1}} < < {1}\;{,}\;{\uptau }_{{2}} < < {1}$ if, ${\text{a}}_{{1}} + {\text{a}}_{{2}} \, < {0}$.

The linear system corresponding to equation (23) is as follows^7^:

$$ \frac{{\text{dx(t)}}}{{{\text{dt}}}} = y $$

$$ \frac{{\text{dy(t)}}}{{{\text{dt}}}} = - \upbeta \,{\text{x}} + {\upmu }\,{\text{y}} $$

The characteristic equation for this system is, $\uplambda^{2} - \uplambda \mu + \upbeta = 0$. which has the roots $\uplambda = \frac{{\mu \pm \sqrt {\mu^{2} - 4\upbeta } }}{4}$. Therefore the fixed point for (22) is asymptotically stable if, $\frac{{{{2(\uptau }}_{{2}} - {\uptau }_{{1}} {)}}}{{{\text{a}}_{{1}} {\uptau }_{{1}}^{{2}} + {\text{a}}_{{2}} {\uptau }_{{2}}^{{2}} }} < 0$ and $\frac{{{2}\left( {{\text{a}}_{{1}} + {\text{a}}_{{2}} } \right)}}{{{\text{a}}_{{1}} {\uptau }_{{1}}^{{2}} + {\text{a}}_{{2}} {\uptau }_{{2}}^{{2}} }} > 0$^7^, so ${\text{a}}_{{1}} {\uptau }_{{1}}^{{2}} + {\text{a}}_{{2}} {\uptau }_{{2}}^{{2}} < 0$ and ${\text{a}}_{{1}} + {\text{a}}_{{2}} \, < {0}$ which is the condition for fixed point in (23) with very small delays.

## Parkinson's disease application

The models in (4) and (23) can be presented as models for Parkinson's disease, from Austin’s results, ${\text{a}}_{{1}}$ and ${\text{a}}_{{2}}$ represents negative or positive feedback, which work to reduce or increase symptoms. Due to the inconsistency of drug-blood–brain barrier crossing, Parkinson's disease patients have medication side effects that can lead to more involuntary movements and a movement disorder^8^. In the case ${\text{a}}_{{1}}$ and ${\text{a}}_{{2}}$ are positive the model in (4) is unstable; this is not compatible with biological systems. The simplest delay differential equation is given by, $\frac{dx}{{dt}} = a\;x(t - \tau )$. The negative or positive sign of the constant $a$ is indicates to the negative or positive feedback^9^. Agiza et al. are studying some models for Parkinson's disease^10^ with the case of positive feedback. We suggest generalizing the model in (23) as a model describing the symptoms of Parkinson's disease with a solution to the problem of positive feedback as follows:

24 $$ \frac{{\text{dx(t)}}}{{{\text{dt}}}} = {\text{ a}}_{{1}} {\text{ x(t}} - {\uptau }_{{1}} {)} + {\text{a}}_{{2}} {\text{ x(t}} - {\uptau }_{{2}} { )} - \left( {\text{x(t)}} \right)^{{2}} \left( {{\text{b}}_{{2}} {\text{x(t}} - {\uptau }_{{2}} { )} + {\text{b}}_{{1}} {\text{x(t}} - {\uptau }_{{1}} { )}} \right) $$

where ${\text{a}}_{{1}}$, ${\text{a}}_{{2}}$ , ${\text{b}}_{{1}}$ and ${\text{b}}_{{2}}$ are scalar parameters, ${\text{b}}_{{1}} {\text{b}}_{{2}} < 0$ and $\left| {{\text{b}}_{{1}} } \right| \to \left| {{\text{b}}_{{2}} } \right|$.

In this model there are two fixed points: zero fixed point and non-zero fixed point ${\text{x}}^{*} = \sqrt {\frac{{{\text{a}}_{{1}} + {\text{a}}_{{2}} }}{{{\text{b}}_{{1}} + {\text{b}}_{{2}} }}}$, where $\frac{{{\text{a}}_{{1}} + {\text{a}}_{{2}} }}{{{\text{b}}_{{1}} + {\text{b}}_{{2}} }} > 0$.

For non-zero fixed point the associated linear model for (24) is

25 $$ \frac{{\text{dx(t)}}}{{{\text{dt}}}} = \, \delta {\text{ x(t}} - {\uptau }_{{1}} { )} - \delta {\text{ x(t}} - {\uptau }_{{2}} { )} - 2\left( {{\text{a}}_{{1}} + {\text{a}}_{{2}} } \right){\text{x(t)}} $$

where $$\updelta = \frac{{{\text{a}}_{{1}} {\text{b}}_{{2}} - {\text{a}}_{{2}} {\text{b}}_{{1}} }}{{{\text{b}}_{{2}} - 1}}$$.

Characteristic equation is given by

26 $$ \uplambda = \updelta {\text{e}}^{{ - {{\uplambda \uptau }}_{{1}} }} - \updelta {\text{e}}^{{ - {{\uplambda \uptau }}_{{2}} }} - {\text{2(a}}_{{1}} + {\text{a}}_{{2}} {)} $$

For negative ${\uplambda }$ and delays ${\uptau }_{{2}} > {\uptau }_{{1}}$

27 $$ {\uplambda } = - 2({\text{a}}_{{1}} + {\text{a}}_{{2}} ) $$

Then non-zero fixed point ${\text{x}}^{*} = \sqrt {\frac{{{\text{a}}_{{1}} + {\text{a}}_{{2}} }}{{{\text{b}}_{{1}} + {\text{b}}_{{2}} }}}$ is asymptotically stable if,

28 $$ {\text{a}}_{{1}} + {\text{a}}_{{2}} \, > {0} $$

If the negative feedback is greater than the positive feedback, then symptoms will end. If the positive feedback is greater than the negative feedback, then symptoms will not disappear, but will stabilize at the non-zero stability point.

## Numerical cases studies

**Case 1:** $\frac{{{\text{dx}}}}{{{\text{dt}}}} = - {\text{10 x(t}} - 0.{2 )} + 3{\text{ x(t}} - 0.{3 )}$, where ${\text{x(t)}} = {2 }$ for ${\text{t}} \le {0}$.

**Case 2:** $\frac{{{\text{dx}}}}{{{\text{dt}}}} = - {\text{3x(t}} - 0.{1 )} - {\text{5 x(t}} - 0.{3 )}$, where ${\text{x(t)}} = {2 }$ for ${\text{t}} \le {0}$.

**Case 3:** $\frac{{{\text{dx}}}}{{{\text{dt}}}} = - {\text{7 x}}\left( {{\text{t}} - \frac{\uppi }{{{20}}}} \right) + 3{\text{x}}\left( {{\text{t}} - \frac{3\uppi }{{{20}}}} \right)$, where ${\text{x(t)}} = {2 }$ for ${\text{t}} \le {0}$.

**Case 4:** $\frac{{{\text{dx}}}}{{{\text{dt}}}} = - {0}{\text{.9 x}}\left( {{\text{t}} - \frac{\uppi }{{2}}} \right) + 0.2{\text{x}}\left( {{\text{t}} - \frac{3\uppi }{{2}}} \right) - {\text{x}}^{{2}} \left[ {{\text{x}}\left( {{\text{t}} - \frac{3\uppi }{{2}}} \right) - {\text{x}}\left( {{\text{t}} - \frac{\uppi }{{2}}} \right)} \right]$, where ${\text{x(t)}} = {0}.4$ for ${\text{t}} \le {0}$.

**Case 5:** $\frac{{{\text{dx}}}}{{{\text{dt}}}} = - {1}{\text{.95 x(t}} - \uppi {)} + 2.5{\text{x}}\left( {{\text{t}} - \frac{3\uppi }{{2}}} \right) - {\text{x}}^{{2}} \left[ {{1}{\text{.55x}}\left( {{\text{t}} - \frac{3\uppi }{{2}}} \right) - {\text{x(t}} - \uppi { )}} \right]$, where ${\text{x(t)}} = 1.1$ for ${\text{t}} \le {0}$.

**Case 6:** $\frac{{{\text{dx}}}}{{{\text{dt}}}} = - {\text{8 x(t}} - 0.{1 )} + 4{\text{x(t}} - {5)} - {\text{x}}^{{2}} \left[ {{\text{x(t}} - 5{ )} - {\text{x(t}} - {0}{\text{.1)}}} \right]$, where ${\text{x(t)}} = 2$ for ${\text{t}} \le {0}$.

**Case 7:** $\frac{{{\text{dx}}}}{{{\text{dt}}}} = - {\text{14 x(t}} - 0.{1 )} + 2{\text{x(t}} - {5 )} - {\text{x}}^{{2}} \left[ {{\text{x(t}} - 5{ )} - {\text{x(t}} - {0}{\text{.1)}}} \right]$, where ${\text{x(t)}} = 2$ for ${\text{t}} \le {0}$.

**Case 8:** $\frac{{{\text{dx}}}}{{{\text{dt}}}} = {\text{3 x(t}} - 0.{1 )} - 4{\text{x(t}} - {5 )} + {\text{x}}^{{2}} \left[ {{1}{\text{.1x(t}} - 5{ )} - {\text{x(t}} - {0}{\text{.1)}}} \right]$, where ${\text{x(t)}} = 2$ for ${\text{t}} \le {0}$.

Case 9: $\frac{{{\text{dx}}}}{{{\text{dt}}}} = - {\text{6 x(t}} - 0.{1 )} + {\text{4x(t}} - {0}{\text{.3 )}} + {\text{x}}^{{2}} \left[ {{1}{\text{.1x(t}} - 5{ )} - {\text{x(t}} - {0}{\text{.1)}}} \right]$, where ${\text{x(t)}} = 2$ for ${\text{t}} \le {0}$.

**Case 10:** $\frac{{{\text{dx}}}}{{{\text{dt}}}} = {0}{\text{.2 x(t}} - 0.{1 )} + {0}.1{\text{x(t}} - {0}{\text{.3 )}} + {\text{x}}^{{2}} \left[ {{1}{\text{.1x(t}} - 5{ )} - {\text{x(t}} - {0}{\text{.1)}}} \right]$, where ${\text{x(t)}} = 1$ for ${\text{t}} \le {0}$.

**Figure 1:**
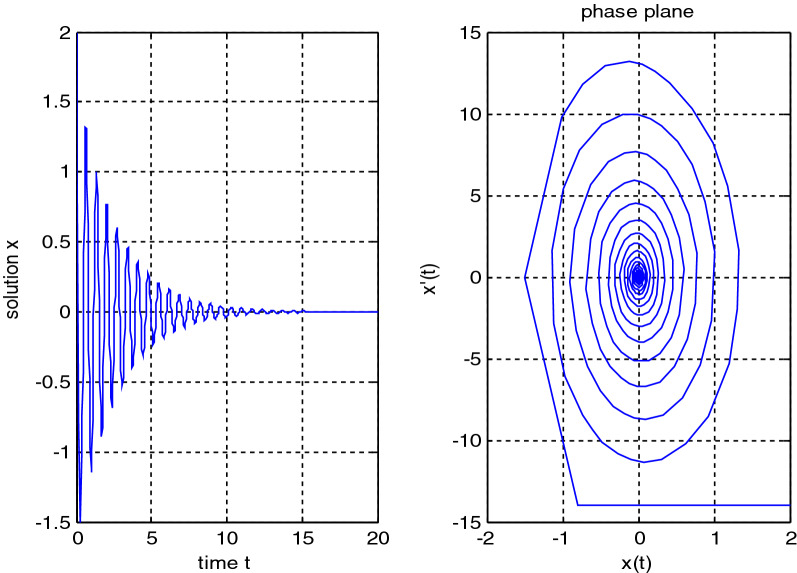
Delay differential equation $\frac{{{\text{dx}}}}{{{\text{dt}}}} = {\text{a}}_{{1}} {\text{ x(t}} - {\uptau }_{{1}} { )} + {\text{a}}_{{2}} {\text{ x(t}} - {\uptau }_{{2}} { )}$ as damped harmonic oscillator.

**Figure 2:**
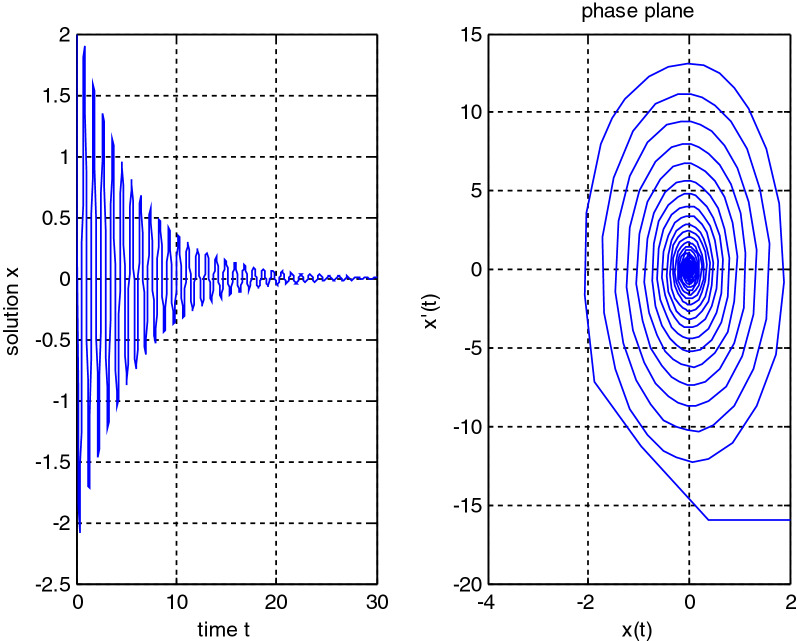
Delay differential equation $\frac{{{\text{dx}}}}{{{\text{dt}}}} = {\text{a}}_{{1}} {\text{ x(t}} - {\uptau }_{{1}} { )} + {\text{a}}_{{2}} {\text{ x(t}} - {\uptau }_{{2}} { )}$, ${\text{a}}_{{1}} < 0,\;{\text{a}}_{2} < 0$.

**Figure 3:**
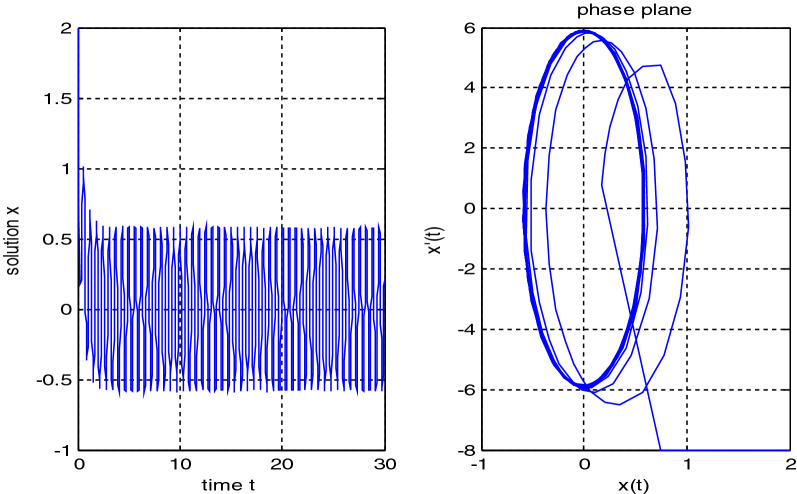
Limit cyclic for Eq. $\frac{{{\text{dx}}}}{{{\text{dt}}}} = {\text{a}}_{{1}} {\text{ x(t}} - {\uptau }_{{1}} { )} + {\text{a}}_{{2}} {\text{ x(t}} - {\uptau }_{{2}} { )}$.

**Figure 4:**
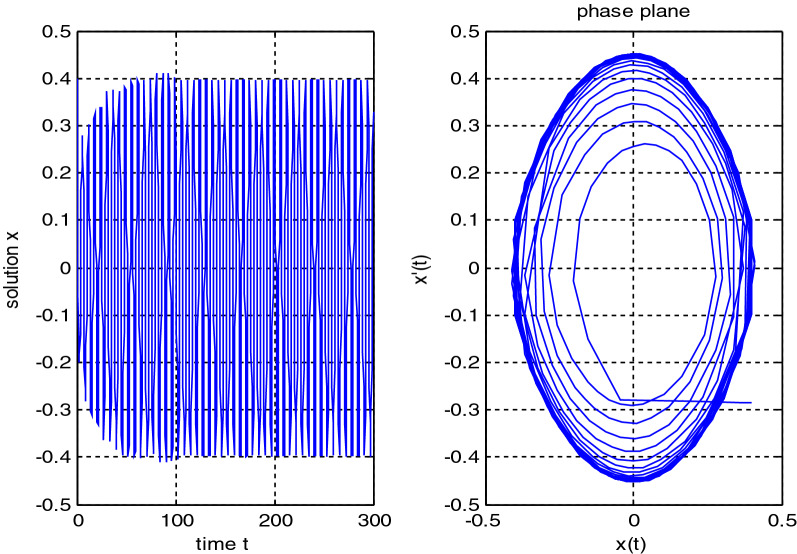
Limit cyclic for Eq. $\frac{{\text{dx(t)}}}{{{\text{dt}}}} = {\text{ a}}_{{1}} {\text{ x(t}} - {\uptau }_{{1}} { )} + {\text{a}}_{{2}} {\text{ x(t}} - {\uptau }_{{2}} { )} - \left( {\text{x(t)}} \right)^{{2}} \left( {{\text{x(t}} - {\uptau }_{{2}} { ) } - {\text{ x(t}} - {\uptau }_{{1}} { )}} \right)$.

**Figure 5:**
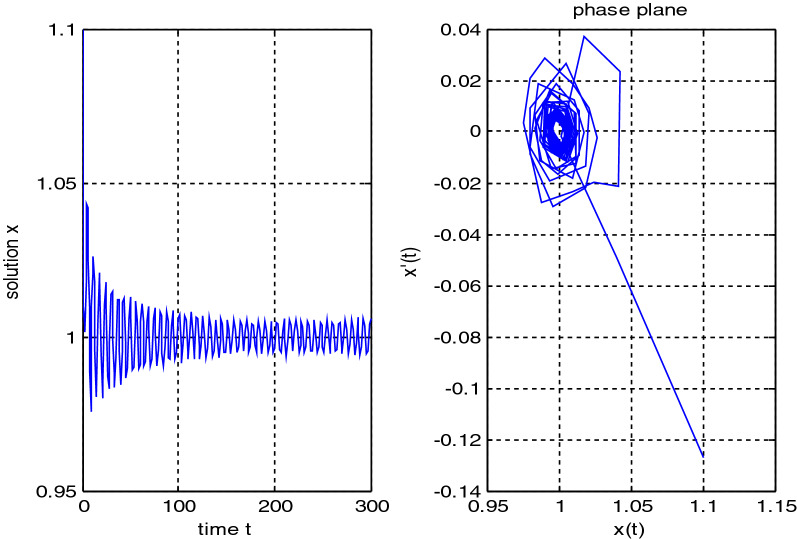
Limit cyclic for Eq. $\frac{{\text{dx(t)}}}{{{\text{dt}}}} = {\text{ a}}_{{1}} {\text{ x(t}} - {\uptau }_{{1}} { )} + {\text{a}}_{{2}} {\text{ x(t}} - {\uptau }_{{2}} { )} - \left( {\text{x(t)}} \right)^{{2}} \left( {{\text{b}}_{{2}} {\text{x(t}} - {\uptau }_{{2}} { )} + {\text{b}}_{{1}} {\text{x(t}} - {\uptau }_{{1}} { )}} \right)$.

**Figure 6:**
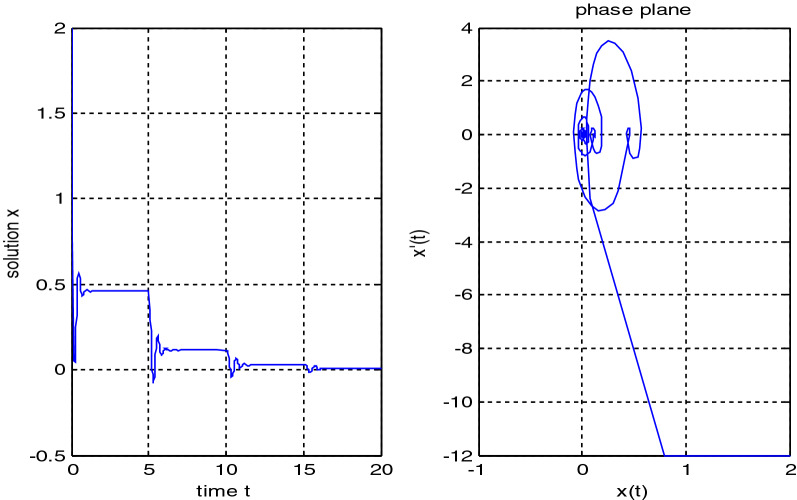
$\tau_{1} = 0.{1 }$ Small delay and $\tau_{2} = 5 \,$ large delay for Eq. $\frac{{\text{dx(t)}}}{{{\text{dt}}}} = {\text{ a}}_{{1}} {\text{ x(t}} - {\uptau }_{{1}} { )} + {\text{a}}_{{2}} {\text{ x(t}} - {\uptau }_{{2}} { )} - \left( {\text{x(t)}} \right)^{{2}} \left( {{\text{x(t}} - {\uptau }_{{2}} { )} - {\text{ x(t}} - {\uptau }_{{1}} { )}} \right)$ with ${\text{a}}_{{1}} = - {\text{8 , a}}_{{2}} = {4}$.

**Figure 7:**
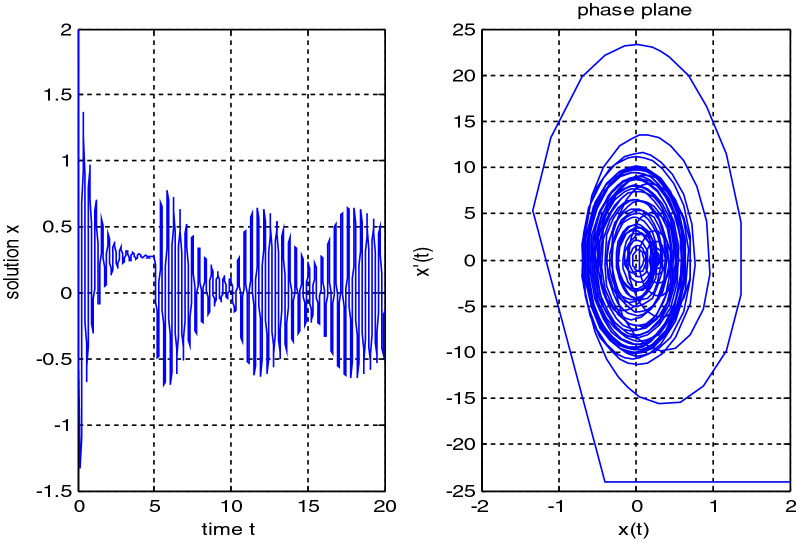
$\tau_{1} = 0.{1 }$ Small delay and $\tau_{2} = 5 \,$ large delay for Eq. $\frac{{\text{dx(t)}}}{{{\text{dt}}}} = {\text{ a}}_{{1}} {\text{ x(t}} - {\uptau }_{{1}} { )} + {\text{a}}_{{2}} {\text{ x(t}} - {\uptau }_{{2}} { )} - \left( {\text{x(t)}} \right)^{{2}} \left( {{\text{x(t}} - {\uptau }_{{2}} { )} - {\text{ x(t}} - {\uptau }_{{1}} { )}} \right)$ with ${\text{a}}_{{1}} = - {\text{14 , a}}_{{2}} = {2}$.

**Figure 8:**
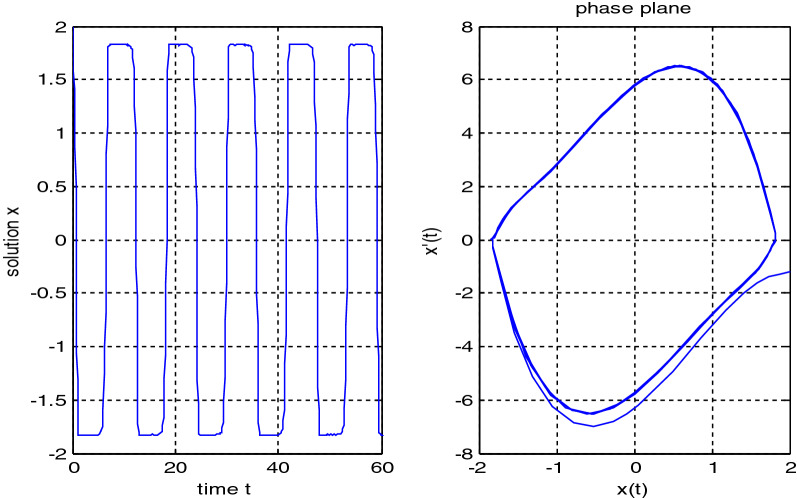
Relaxation oscillation for Eq. $\frac{{\text{dx(t)}}}{{{\text{dt}}}} = {\text{ a}}_{{1}} {\text{ x(t}} - {\uptau }_{{1}} { )} + {\text{a}}_{{2}} {\text{ x(t}} - {\uptau }_{{2}} { )} - \left( {\text{x(t)}} \right)^{{2}} \left( {{\text{b}}_{{2}} {\text{x(t}} - {\uptau }_{{2}} { )} + {\text{b}}_{{1}} {\text{x(t}} - {\uptau }_{{1}} { )}} \right)$ with ${\text{a}}_{{1}} = {\text{3 , a}}_{{2}} = { - }4$.

**Figure 9:**
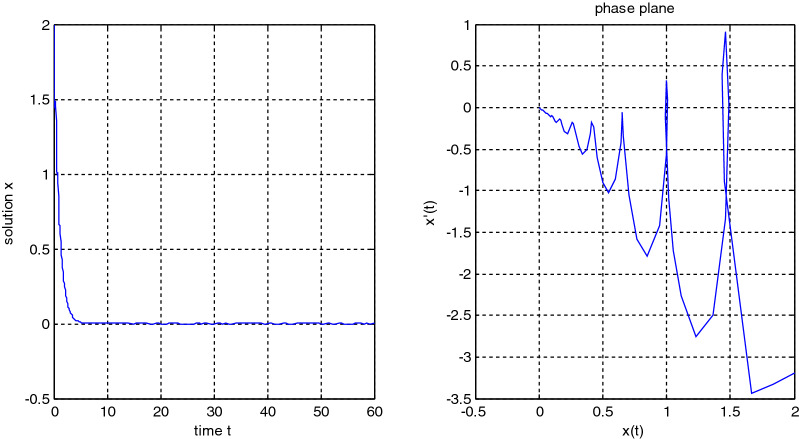
Parkinson's model zero fixed point with stability condition ${\text{a}}_{{1}} + {\text{a}}_{{2}} < 0$.

**Figure 10:**
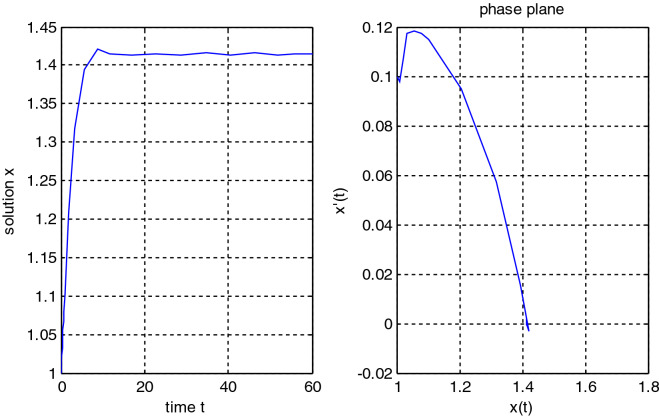
Parkinson's model non-zero fixed point = $\sqrt 2$ with stability condition ${\text{a}}_{{1}} + {\text{a}}_{{2}} < 0$.

In the previous numerical examples, the Matlab program was used to solve delay differential equations using the code dde23. The numerical simulation showed many cases of the proposed equations. Numerical simulation for the equation $\frac{{{\text{dx}}}}{{{\text{dt}}}} = {\text{a}}_{{1}} {\text{ x(t}} - {\uptau }_{{1}} { )} + {\text{a}}_{{2}} {\text{ x(t}} - {\uptau }_{{2}} { )}$ showed the damped harmonic oscillation with different values of parameters ${\text{a}}_{{1}}$ and ${\text{a}}_{{2}}$ in Figs. 1 and 2, also the Hopf bifurcation case in Fig. 3. In Figs. 4, 5, 6 and 7 different cases that can be expressed by the equation $\frac{{\text{dx(t)}}}{{{\text{dt}}}} = {\text{ a}}_{{1}} {\text{ x(t}} - {\uptau }_{{1}} { )} + {\text{a}}_{{2}} {\text{ x(t}} - {\uptau }_{{2}} { )} - \left( {\text{x(t)}} \right)^{{2}} \left( {{\text{x(t}} - {\uptau }_{{2}} {)} - {\text{ x(t}} - {\uptau }_{{1}} {)}} \right)$. In Fig. 8, the numerical simulation showed a case of relaxation oscillation. In Fig. 9, the feedback is positive and negative, and the fixed point is zero, so symptoms disappear. In Fig. 10, the feedback is positive and the fixed point is non-zero, and symptoms stabilize at the non-zero fixed point for Parkinson's disease.

## Conclusion

Van der Pol model has many applications, especially neurological applications. Van der Pol model is used to describe the defects in Parkinson's disease, and a mathematical model for this disease is made using the delay differential equations in^4^.

From the results of the study by Austin in^3^, there are factors that work to reduce the defect, and thus act as negative feedback, and other factors that increase the defect and thus act as positive feedback. The model can then be assumed using the linear delay differential equations in (4), which ${\text{ a}}_{{1}}$ act as negative feedback for control on delay time $\tau_{{1}}$ and ${\text{ a}}_{{2}}$ act as positive feedback for control off delay time $\tau_{{2}}$.

In this paper, the differential equation of the Van der Pol model is deduced from a delay differential equation with two small delays, which opens the way for the re-use of the applications of the Van der Pol model by using differential equations with two delays.

In Parkinson's disease, the side effects of medication appear after a period of time, by giving them, and the patient's response to treatment decreases, and it may work to increase motor symptoms. A model has been modified to comply in this case.

Some numerical cases of the LRC circuit model have been simulated. See Figs. 1 and 2. Van der Pol model has been simulated. See Figs. 4, 5, 6, 7 and 8, limit cyclic appears in Figs. 3, 4 and 5, relaxation oscillation appears in Figs. 7 and 8, the effect of the non-linear part appears whenever the coefficients of the linear part are small. In the case of a small delay and a large delay, a repetition occurs and a relaxation oscillation occurs.

Van der Pol created a series of electrical circuit models of the human heart to investigate the spectrum of cardiac dynamics' stability. His experiments with adding an external driving signal were similar to a pacemaker driving a genuine heart. He wanted to learn how to control an erratic heartbeat^2^. The derivation of the Van der Pol equation as a delay differential equation with two delays can have an impact on Van der Pol applications such as the pacemaker. The proposed Eq. (24) is more dynamic and comprehensive and needs another study into the conditions of chaos, if any, and the conditions for Hopf bifurcation.
